# High-throughput volumetric adaptive optical imaging using compressed time-reversal matrix

**DOI:** 10.1038/s41377-021-00705-4

**Published:** 2022-01-14

**Authors:** Hojun Lee, Seokchan Yoon, Pascal Loohuis, Jin Hee Hong, Sungsam Kang, Wonshik Choi

**Affiliations:** 1 Center for Molecular Spectroscopy and Dynamics, Institute for Basic Science, Seoul, 02841 Korea; 2 Department of Physics, Korea University, Seoul, 02841 Korea; 3 Department of Applied Mathematics, University of Twente, Drienerlolaan 5, 7522 NB Enschede, Netherlands; 4 Achmea Holding BV, Handelsweg 2, 3707 NH Zeist, Netherlands

**Keywords:** Adaptive optics, Interference microscopy, Imaging and sensing

## Abstract

Deep-tissue optical imaging suffers from the reduction of resolving power due to tissue-induced optical aberrations and multiple scattering noise. Reflection matrix approaches recording the maps of backscattered waves for all the possible orthogonal input channels have provided formidable solutions for removing severe aberrations and recovering the ideal diffraction-limited spatial resolution without relying on fluorescence labeling and guide stars. However, measuring the full input–output response of the tissue specimen is time-consuming, making the real-time image acquisition difficult. Here, we present the use of a time-reversal matrix, instead of the reflection matrix, for fast high-resolution volumetric imaging of a mouse brain. The time-reversal matrix reduces two-way problem to one-way problem, which effectively relieves the requirement for the coverage of input channels. Using a newly developed aberration correction algorithm designed for the time-reversal matrix, we demonstrated the correction of complex aberrations using as small as 2% of the complete basis while maintaining the image reconstruction fidelity comparable to the fully sampled reflection matrix. Due to nearly 100-fold reduction in the matrix recording time, we could achieve real-time aberration-correction imaging for a field of view of 40 × 40 µm^2^ (176 × 176 pixels) at a frame rate of 80 Hz. Furthermore, we demonstrated high-throughput volumetric adaptive optical imaging of a mouse brain by recording a volume of 128 × 128 × 125 µm^3^ (568 × 568 × 125 voxels) in 3.58 s, correcting tissue aberrations at each and every 1 µm depth section, and visualizing myelinated axons with a lateral resolution of 0.45 µm and an axial resolution of 2 µm.

## Abstract (web summary)

Compressed time-reversal matrix microscopy was developed for high-throughput volumetric adaptive optical imaging, enabling aberration-free visualization of myelinated axons in a mouse brain with high volumetric resolution.

## Graphical abstract

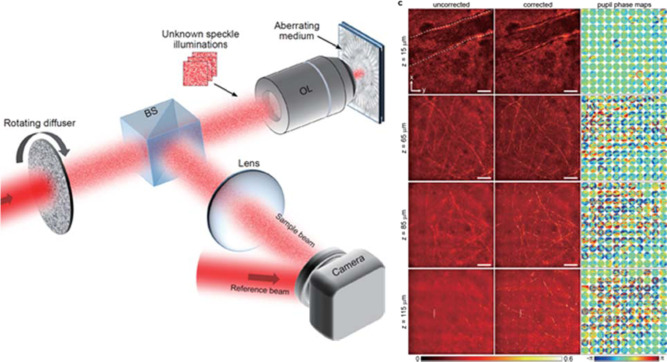

## Introduction

An arbitrary optical system interacting with light waves can be described by transmission and reflection matrices, as far as the linear light-matter interaction is concerned. The transmission (reflection) matrix of an optical system describes the transmitted (reflected) electric field (E-field) at all the possible detection channels for a set of orthogonal illumination channels. Due to the characterization of the input–output response, the measured matrix can be considered as a replica of a real optical system within the context of the covered illumination/detection channels. Therefore, one can computationally process it as though a real experiment is being conducted. The knowledge of the matrix allows one to find solutions in a variety of applications where lengthy experimental optimizations are required. Examples include focusing light^1,2^, delivering images^3^, and controlling transmission power^4^ through scattering media based on the transmission matrix. The reflection matrix, suitable for more realistic in vivo applications for which the detector cannot be placed on the transmission side, has provided exceptional opportunities for deep-tissue imaging^5^. The reflection matrix has also been exploited to focus light on a target embedded deep within strongly scattering media^6–8^. The wave correlation of the single-scattered waves in the reflection matrix was tailored to attenuate the effect of multiple light scattering^9^. A wavefront correction algorithm termed closed-loop accumulation of single scattering (CLASS)^10^ was developed based on the time-gated reflection matrix for separately identifying the aberrations in the illumination and detection pathways without the need for guide stars and in the presence of strong multiple scattering noise. This offers imaging deep within biological tissues with a subdiffraction-limited resolution^11^. The singular value decomposition (SVD) was applied to a time-gated reflection matrix for retrieving a target image underneath strongly scattering media^12,13^. Recently, it has been demonstrated that the time-gated reflection matrix measured in the space domain made it possible to image a mouse brain through an intact skull inducing extreme aberrations^14^. Indeed, the reflection matrix approaches provide formidable solutions in the context of computational adaptive optics (AO) microscopy^15,16^ in that they can deal with extremely severe aberrations with no need for fluorescence labeling and guide stars. In addition, this space-domain reflection matrix study proved that it can serve as a type of wavefront sensorless AO^17,18^ that is combined with hardware correction of aberration by wavefront shaping devices such as a spatial light modulator and deformable mirror to realize ideal diffraction-limited multi-photon fluorescence imaging through an intact skull^14^.

Despite these benefits, the matrix-based AO approach has been elusive in real-time bio-medical imaging applications. The recording of the full reflection matrix is a time-consuming process because the E-field map of the reflected wave must be measured for each illumination channel, as opposed to confocal imaging’s requirement of point detection. Furthermore, the interferometric detection of the E-field is sensitive to the random phase drift, which can deteriorate the recorded reflection matrix in the dynamic samples. Sparse sampling of the matrix would be a potential solution, but this is accompanied by incomplete sampling of illumination channels. Considering that finding object information embedded within a scattering medium requires identifications of wave distortions in both the illumination and detection pathways, insufficient sampling of the illumination channels can undermine the capability to resolve illumination distortions.

To overcome these issues, we consider a time-reversal matrix ${\boldsymbol{RR}}^\dagger$ instead of the reflection matrix ${\boldsymbol{R}}$. Here ${\boldsymbol{R}}^\dagger$ represents the conjugate transpose of ${\boldsymbol{R}}$. Unlike the reflection matrix itself, which describes the relationship between the illumination and detection channels, the time-reversal matrix describes the phase-conjugated roundtrip process connecting the detection channels to the same detection channels via the illumination channels. Essentially, this reduces the two-way problem with the reflection matrix to the one-way problem with the time-reversal matrix on condition that the illumination channels are orthogonal. There are two major benefits of dealing with the time-reversal matrix. It can maintain high fidelity in terms of retaining the information on the detection channels even if the illumination channel coverage is much smaller than that of the complete set. Furthermore, it is not even necessary to know the basis of the illumination channels, making it robust to the random phase drift.

Here, we present a high-throughput volumetric AO imaging method termed a compressed time-reversal closed-loop accumulation of single scattering (CTR-CLASS), in which the previously developed CLASS algorithm was extended to a compressed time-reversal matrix constructed by a sparsely sampled reflection matrix for correcting the complex sample-induced aberrations with significantly reduced number of measurements. In this implementation, we took advantage of the time-reversal matrix and made use of dynamically varying unknown speckle patterns as illumination channels. We demonstrated that both the aberration map and object image can be retrieved using the number of speckle patterns as small as 2% of the complete basis while maintaining comparable fidelity to that of the fully sampled matrix. Due to nearly 100-fold reduction of the matrix recording time, the CTR-CLASS has enabled real-time aberration-correction imaging for a field of view (FOV) of 40 × 40 µm^2^ (176 × 176 pixels) at a frame rate of 80 Hz. We applied the developed method for the volumetric AO imaging of ex vivo mouse brain and visualized myelinated axons with a lateral resolution of 0.45 µm and axial resolution of 2 µm over a volume of 128 × 128 × 125 µm^3^ (568 × 568 × 125 voxels) within a recording time of 3.58 s.

## Results

### Reflection matrix description of an imaging system

**Fig. 1:**
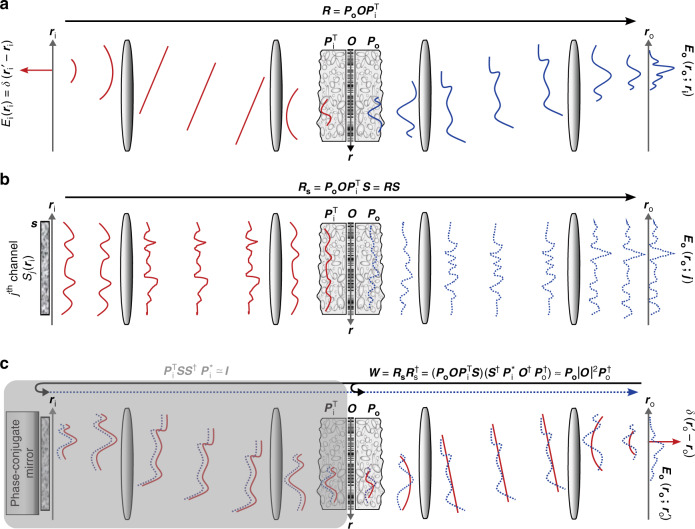
Schematics of imaging geometry and time-reversal process. **a** Description of the reflection matrix ***R*** in point illumination basis. ${\boldsymbol{R}}[{{\bf{r}}}_{\rm{o}};{{\bf{r}}}_{\rm{i}}^{{\prime}}]=[{E}_{\rm{o}}({{\bf{r}}}_{\rm{o}};{{\bf{r}}}_{\rm{i}}^{{\prime}})]$ is a set of E-field responses ${{E}}_{\rm{o}}({{\bf{r}}}_{\rm{o}};{{\bf{r}}}_{\rm{i}}^{\prime })$ to impulse input fields ${E}_{\rm{i}}({{\bf{r}}}_{\rm{i}})=\delta ({{\bf{r}}}_{\rm{i}}-{{\bf{r}}}_{\rm{i}}^{{\prime} })$. For clarity, the reflection pathway is unfolded to the transmission side. ***O*** is the object’s reflection coefficient matrix, and ${{\boldsymbol{P}}}_{\rm{i(o)}}=[{P}_{\rm{i(o)}}({\bf{r}};{{\bf{r}}}_{\rm{i(o)}})]$ is the transmission matrix of the scattering medium for wave propagation from position **r** to ${{\bf{r}}}_{\rm{i(o)}}$. The red and blue curves represent incident and reflected waves, respectively. **b** Reflection matrix ***R***_S_ in the case of speckle illumination. ${{\boldsymbol{R}}}_{\rm{S}}[{{\bf{r}}}_{\rm{o}};j]=[{E}_{\rm{o}}({{\bf{r}}}_{\rm{o}};j)]$ is a set of E-field responses ${E}_{\rm{o}}({{\bf{r}}}_{\rm{o}};j)$ for speckle input channels $S({{\bf{r}}}_{\rm{i}};j)$. ***R***_S_ can be written as ***R***_S_ = ***RS***, where ${\boldsymbol{S}}[{{\bf{r}}}_{\rm{i}};j]=[S({{\bf{r}}}_{\rm{i}};j)]$ is the input illumination matrix. **c** Geometric interpretation of the time reversal matrix. The time-reversal matrix ${\boldsymbol{W}}[{{\bf{r}}}_{\rm{o}};{{\bf{r}}}_{\rm{o}}^{{\prime} }]={{\boldsymbol{R}}}_{\rm{S}}{{\boldsymbol{R}}}_{\rm{S}}^{\dagger }$ can be interpreted as a reflection matrix describing a roundtrip process for a wave propagating from the output plane **r**_o_ to the object plane **r** and reflecting back to the output plane by the target object |***O***|^2^

Let us first start with a mathematical model for an optical imaging system of interest using reflection matrix formalism. We consider the time-gated coherent imaging of a target object through a scattering medium in reflection geometry (Fig. 1a). For convenience, the optical layout is unfolded by flipping the reflection beam path over an object plane, making the layout analogous to transmission geometry. Since the scattering sample serves as a linear system with respect to the E-field in coherent imaging, the reflected wave can be described by a linear superposition of impulse response functions,

1 $${E}_{{\rm{o}}}({{\bf{r}}}_{{\rm{o}}};{{\bf{r}}}_{{\rm{i}}})=\int {P}_{{\rm{o}}}({{\bf{r}}}_{{\rm{o}}};{\bf{r}})O({\bf{r}}){P}_{{\rm{i}}}({{\bf{r}}}_{{\rm{i}}};{\bf{r}}){d}^{2}{\bf{r}}+{E}_{{\rm{ms}}}({{\bf{r}}}_{{\rm{o}}};{{\bf{r}}}_{{\rm{i}}})$$

Here, *E*_o_ (**r**_o_**;r**_i_) is the time-gated E-field at position **r**_o_ on the output (detection) plane when a target object is illuminated by a point source located at position **r**_i_ on the input (illumination) plane. *O*(**r**) is the object function that represents complex reflection coefficients of the target object. Both the input and output planes are conjugate to the object plane whose spatial coordinate is **r**. *P*_i(o)_ (**r**_i(o)_; **r**) is the time-gated E-field point-spread-function (PSF) that represents E-field distribution at the input (output) plane generated by a point source located at a position **r** on the object plane. The PSF describes transmissions of ballistic waves that maintain their propagation directions in propagation through the scattering medium. Therefore, it has the linear shift-invariant property^19^, i.e., ${P}_{{\rm{i}}({\rm{o}})}({{\bf{r}}}_{{\rm{i}}({\rm{o}})};{\bf{r}})={P}_{{\rm{i}}({\rm{o}})}({{\bf{r}}}_{{\rm{i}}({\rm{o}})}-{\bf{r}})$. *E*_ms_ (**r**_o_; **r**_i_) represents speckle noise generated by multiple-scattered waves in the scattering medium, whose flight times fall within a finite time-gating window. In scattering matrix formalism, Eq. (1) can be represented by a time-gated reflection matrix ***R*** whose element is *E*_o_ (**r**_o_; **r**_i_) for a column index **r**_i_ and row index **r**_o_. Based on Eq. (1), ***R*** can be decomposed as

2 $${\boldsymbol{R}}={{\boldsymbol{P}}}_{{\rm{o}}}{\boldsymbol{O}}{{\boldsymbol{P}}}_{{\rm{i}}}^{{\rm{T}}}+{{\boldsymbol{R}}}_{{\rm{ms}}}$$

Here, ***O*** is a diagonal matrix whose diagonal element is *O*(**r**). ***P***_i_ and ***P***_o_ are Toeplitz (diagonal-constant) matrices whose elements are respectively given by the input and output PSFs, *P*_i_ (**r**_i_; **r**) and *P*_o_ (**r**_o_; **r**). ***R***_ms_ is a matrix composed of *E*_ms_ (**r**_o_; **r**_i_), and the superscript ‘T’ denotes matrix transpose operation. The first term on the right-hand side in Eq. (2) is responsible for image reconstruction. Based on the perturbation of the first-order Born approximation, the first term assumes that the incident wave does not change its propagation direction until it reaches the object plane, but does experiences phase retardation by the scattering medium. For identical illumination and detection paths, input and output PSFs are the same due to the reciprocity principle in optics and thus satisfy the relation ${P}_{{\rm{i}}}({\bf{r}}^{\prime} ;{\bf{r}})={P}_{{\rm{o}}}({\bf{r}}^{\prime} ;{\bf{r}})$. However, this is not a necessary condition in the present study. As we reported earlier^10^, the CLASS algorithm utilizing ***R*** is developed in such a way to find high-fidelity solutions for an unknown object function (***O***) and two PSFs (***P***_i_ and ***P***_o_) even in the presence of strong multiple scattering noise ***R***_ms_.

### Compressive sensing of the reflection matrixS

The matrix ***R*** in Eq. (2) can be directly measured by scanning the position **r**_i_ of the focused illumination and wide-field detection of the backscattered wave field ${E}_{{\rm{o}}}({{\bf{r}}}_{{\rm{o}}};{{\bf{r}}}_{{\rm{i}}})$ across **r**_o_. To obtain the full time-gated reflection matrix for a given FOV, it is necessary to scan a focused beam over the FOV with a lateral sampling interval of the diffraction-limited resolution, *Δx* = *λ*/(2NA), where *λ* is the wavelength of light source, and NA is the objective numerical aperture. For a 2-dimensional (2D) FOV of size *L* × *L*, the required number of sampling points for a complete sampling is given by *N* = *L*/*Δx* which is the total number of orthogonal spatial modes for the given FOV, NA, and *λ*. In fact, the full reflection matrix ${\boldsymbol{R}}\in {{\Bbb{C}}}^{N\times N}$ can be measured by sending any complete set ${\{{E}_{{\rm{i}}}({\bf{r}}_{\rm{i}};j)\}}_{j=1}^{N}$ of *N* illumination fields, instead of point-by-point scanning with a focused beam. One can measure the respective output field ${E}_{{\rm{o}}}({{\bf{r}}}_{{\rm{o}}};j)$ for each *j*^th^ illumination and construct a reflection matrix ${{\boldsymbol{R}}}_{{\rm{m}}}\in {{\Bbb{C}}}^{N\times N}$ whose columns are assigned by the measured output E-fields ${\{{E}_{{\rm{o}}}({{\bf{r}}}_{{\rm{o}}};j)\}}_{j=1}^{N}$. In this case, the column and row indices of ***R***_m_ are *j* and **r**_o_, respectively. Then, the measured ***R***_m_ is expressed as ${{\boldsymbol{R}}}_{{\rm{m}}}={\boldsymbol{R}}{{\boldsymbol{E}}}_{{\rm{i}}}$, where ${{\boldsymbol{E}}}_{{\rm{i}}}\in {{\Bbb{C}}}^{N\times N}$ is an illumination matrix constructed by ${\{{E}_{{\rm{i}}}({{\bf{r}}}_{{\rm{i}}};j)\}}_{j=1}^{N}$ in the same way as ***R***_m_. The reflection matrix of the sample can be obtained by multiplying the measured matrix ***R***_m_ by the inverse of ***E***_i_, i.e., ${\boldsymbol{R}}={{\boldsymbol{R}}}_{{\rm{m}}}{{\boldsymbol{E}}}_{{\rm{i}}}^{-1}$. This requires knowledge of the illumination fields.

Complete sampling of the reflection matrix for a large FOV can be time-consuming and resource-intensive, and thus limits its practical applicability. Main objective of this study is to reduce the data acquisition time. Here, we propose the use of a set of *M* unknown random speckle illumination patterns, ${\{S({{\bf{r}}}_{{\rm{i}}};j)\}}_{j=1}^{M}$ for compressive sensing of ***R***. In particular, we consider the case in which *M* is significantly smaller than *N* (*M* ≪ *N*). The time-gated E-field image ${E}_{{\rm{o}}}({{\bf{r}}}_{{\rm{o}}};j)$ is recorded for each *j*^th^ speckle illumination, and the sparsely-sampled reflection matrix ${{\boldsymbol{R}}}_{{\rm{S}}}\in {{\Bbb{C}}}^{N\times M}$ is then constructed using ${\{{E}_{{\rm{o}}}({{\bf{r}}}_{{\rm{o}}};j)\}}_{j=1}^{M}$ as a matrix element (Fig. 1b). Therefore, the matrix ***R***_S_ with column index *j* and row index **r**_o_ can be expressed as

3 $${{\boldsymbol{R}}}_{{\rm{s}}}={{\boldsymbol{P}}}_{{\rm{o}}}{\boldsymbol{O}}{{\boldsymbol{P}}}_{{\rm{i}}}^{{\rm{T}}}{\boldsymbol{S}}+{{\boldsymbol{R}}}_{{\rm{ms}}}{\boldsymbol{S}}$$

where ${\boldsymbol{S}}\in {{\mathbb{C}}}^{N\times M}$ is the sensing matrix constructed by ${\{S({{\bf{r}}}_{{\rm{i}}};j)\}}_{j=1}^{M}$. To realize aberration correction and image reconstruction without a prior knowledge of the illumination pattern, we consider a CTR matrix, ${\boldsymbol{W}}={{\boldsymbol{R}}}_{{\boldsymbol{s}}}{{\boldsymbol{R}}}_{{\boldsymbol{s}}}^{\dagger }$. By inserting Eq. (3) in ***W***, the matrix is expressed as ${\boldsymbol{W}}=({{\boldsymbol{P}}}_{{\rm{o}}}{\boldsymbol{O}}{{\boldsymbol{P}}}_{{\rm{i}}}^{{\rm{T}}}{\bf{S}})({{\boldsymbol{S}}}^{\dagger }{{\boldsymbol{P}}}_{{\rm{i}}}^{\ast }{{\boldsymbol{O}}}^{\dagger }{{\boldsymbol{P}}}_{o}^{\dagger })+{{\boldsymbol{W}}}_{{\rm{ms}}}$, where ***W***_ms_ is the noise matrix associated with the multiple scattering ***R***_ms_, and the superscript ‘*’ denotes the complex conjugate. Note that the covariance matrix ***SS***^†^ ∈ ${\Bbb{C}}$^*N*×*N*^ is almost an identity matrix ***I*** for sufficiently large *M*. However, finite overlaps among random speckles can cause non-zero off-diagonal elements, i.e., ***SS***^**†**^ = ***I*** + ***σ***, where ***σ*** denotes the additive complex random noise whose matrix elements $\sigma [{{\bf{r}}}_{{\rm{i}}},{{\bf{r}}}_{{\rm{i}}}^{{\prime} }]$ are given by correlations of two series of random speckle fields illuminating different positions, **r**_i_ and ${{\bf{r}}}_{{\rm{i}}}^{{\prime} }$. Therefore, ***W*** can be expressed as

4 $${\boldsymbol{W}}={{\boldsymbol{P}}}_{{\rm{o}}}{{\boldsymbol{O}}}_{{\rm{I}}}{{\boldsymbol{P}}}_{o}^{\dagger }+{{\boldsymbol{W}}}_{{\rm{ms}}}+{{\boldsymbol{W}}}_{\sigma }$$

where ***O***_I_ denotes |***O***|^2^, a diagonal matrix with its diagonal elements given by the reflectance of the object, |*O*(**r**)|^2^. The first term in Eq. (4) uses the relation ${{\boldsymbol{P}}}_{{\rm{i}}}^{{\rm{T}}}{{\boldsymbol{P}}}_{{\rm{i}}}^{\ast }={{\boldsymbol{P}}}_{{\rm{i}}}{{\boldsymbol{P}}}_{{\rm{i}}}^{\dagger }={\boldsymbol{I}}$, which is valid when ${P}_{{\rm{i}}}({{\bf{r}}}_{{\rm{i}}};{\bf{r}})$ is a shift-invariant PSF induced by a phase-only pupil aberration. The last term ${{\boldsymbol{W}}}_{\sigma }={{\boldsymbol{P}}}_{o}{\boldsymbol{O}}{{\boldsymbol{P}}}_{i}^{T}{\boldsymbol{\sigma }}{{\boldsymbol{P}}}_{i}^{\ast }{{\boldsymbol{O}}}^{\dagger }{{\boldsymbol{P}}}_{o}^{\dagger }$ denotes the sparse sampling-induced noise associated with ***σ***. Statistically, mean amplitude of $\sigma [{{\bf{r}}}_{{\rm{i}}},{{\bf{r}}}_{{\rm{i}}}^{{\prime} }]$ is given by ${\langle |{\sum }_{j=1}^{M}S({{\bf{r}}}_{{\rm{i}}};j){S}^{\ast }({{\bf{r}}}_{{\rm{i}}}^{{\prime} };j)|\rangle }_{{{\bf{r}}}_{{\rm{i}}},{{\bf{r}}}_{{\rm{i}}}^{{\prime} }}=1/\sqrt{M}$, where the bracket notation denotes an average over the variables in the subscript. $S({{\bf{r}}}_{{\rm{i}}};j)$ is normalized such that ${\langle {\sum }_{j=1}^{M}{|S({{\bf{r}}}_{{\rm{i}}};j)|}^{2}\rangle }_{{{\bf{r}}}_{{\rm{i}}}}=1$. Therefore, the magnitude of the matrix elements of ***W***_σ_ becomes smaller as *M* increases, which makes ***W***_σ_ negligible for sufficiently large *M*.

Physical interpretation of the time-reversal matrix ***W*** is given in Fig. 1c. In this discussion, we excluded the noise terms in Eq. (4) to focus our attention more on a successful time-reversal process. The noise degrades the fidelity of the aberration correction and the signal-to-noise ratio (SNR) of the reconstructed image, which will be discussed in detail in “Analysis of image SNR” section. By the successive time-reversal operation ${{\boldsymbol{R}}}_{{\rm{S}}}^{\dagger }$, a spherical wave (red curves) emitted from a point source at a position ${{\bf{r}}}_{{\rm{o}}}^{{\prime} }$ on the output plane propagates in the backward direction through the object (${{\boldsymbol{P}}}_{{\rm{i}}}^{\ast }{{\boldsymbol{O}}}^{\dagger }{{\boldsymbol{P}}}_{{\rm{o}}}^{\dagger }$) followed by a fictious scattering layer whose transmission matrix is ***S***^†^. Afterwards, the ***R***_S_ is applied such that the reflected wave (blue curves) returns to the scattering layer (***S***) and the object (${{\boldsymbol{P}}}_{{\rm{o}}}{\boldsymbol{O}}{{\boldsymbol{P}}}_{{\rm{i}}}^{{\rm{T}}}$) in the forward direction to arrive at the output plane. Here, the important point is that the operation indicated by the shaded gray box (${{\boldsymbol{P}}}_{{\rm{i}}}^{{\rm{T}}}{\boldsymbol{S}}{{\boldsymbol{S}}}^{\dagger }{{\boldsymbol{P}}}_{{\rm{i}}}^{\ast }$) serves as a phase-conjugation mirror when ${\boldsymbol{S}}{{\boldsymbol{S}}}^{\dagger }\approx {\boldsymbol{I}}$, i.e., the illumination speckles are sufficiently orthogonal. In other words, a point source emanating from an object plane comes back to the same position via its travel through ${{\boldsymbol{P}}}_{{\rm{i}}}^{{\rm{T}}}{\boldsymbol{S}}{{\boldsymbol{S}}}^{\dagger }{{\boldsymbol{P}}}_{{\rm{i}}}^{\ast }$. This eliminates the need to consider the input aberration *P*_i_ and illumination patterns **S**. As a result, the matrix ${\boldsymbol{W}}={{\boldsymbol{R}}}_{{\rm{S}}}{{\boldsymbol{R}}}_{{\rm{S}}}^{\dagger }$ can be interpreted as a time-gated reflection matrix describing an imaging system that images a reflective object with the reflectance ***O***_I_ through a scattering medium with an input transmission matrix of ${{\boldsymbol{P}}}_{\rm{o}}^{\ast }$ and an output transmission matrix of ***P***_o_. The whole process becomes an *N*-by-*N* square matrix with its column and row indices both corresponding to **r**_o_.

There are a few major benefits of considering ***W*** instead of ***R***. First, the sensing matrix ***S*** describing the illumination patterns is removed in ***W***, thereby eliminating the need to know the illumination speckle patterns. This makes it possible to send an arbitrary choice of illuminations, such as dynamic speckle patterns generated by a rotating diffuser, and thus it is no longer necessary to scan pre-defined positions of point illumination using scanning mirrors. Second, ***W*** is greatly simplified such that only ***P***_o_ and ***O***_I_ remain to be identified. Imperfection in illumination and detection optics often causes discrepancy between ***P***_i_ and ***P***_o_ in the reflection geometry. ***P***_i_ and ***P***_o_ are intrinsically different in the transmission geometry. However, in ***W***, it is not necessary to consider ***P***_i_. Another critical benefit is the possibility of sparse sampling. The condition ***SS***^†^ **≈** ***I*** satisfies even when *M* is extremely small. In contrast, if there is significant downsampling in the focused illumination, both the ability to identify the aberration and the imaging fidelity are significantly degraded.

### Image reconstruction

The concept of the time-reversal matrix was initially introduced for selective focusing in acoustics^20–22^ and then has been intensively studied in microwaves^23,24^ and optics^7,12,25^. In these previous studies, either iterative operation or the singular value decomposition (SVD) of the time-reversal matrix ***W*** = ***R***^†^***R*** was used for selective focusing on a few highly reflecting targets embedded in a scattering medium. Each input singular vector with a nonzero singular value of ***W*** corresponds to a specific wavefront of the incident light focusing on one of the targets whose reflectivity is proportional to the eigenvalue. This approach has also been applied to deep optical imaging of an extended target in a scattering medium, where the image is reconstructed by using the dominant singular values and corresponding singular vectors^12,13^.

In contrast, we consider the CTR matrix ${\boldsymbol{W}}={{\boldsymbol{R}}}_{{\rm{S}}}{{\boldsymbol{R}}}_{{\rm{S}}}^{\dagger }$ and introduce a matrix decomposition of ***W*** into a product of three matrices, ${\boldsymbol{W}}={{\boldsymbol{P}}}_{{\rm{o}}}{{\boldsymbol{O}}}_{{\rm{I}}}{{\boldsymbol{P}}}_{o}^{\dagger }$ in order to find the unknown object function (***O***_I_) embedded within a scattering medium inducing optical aberrations (***P***_o_). In conventional compressive sensing^26,27^, the sampling process is modeled as **y** = ***S*a** + **n**, where ${\bf{y}}\in {{\Bbb{R}}}^{M\times 1}$ is a vector of sparsely sampled data, ${\bf{a}}\in {{\Bbb{R}}}^{N\times 1}$ is the original signal to be recovered, ${\boldsymbol{S}}\in {{\Bbb{R}}}^{M\times N}$ is a known sensing (or measurement) matrix with *M* ≪ *N*, and **n** denotes a measurement noise. Here, *M* is the number of measurements and *N* is the number of signals of interest. The problem of recovering the signal **a** is underdetermined or ill-posed because there are more unknowns than equations. To reliably solve the problem, we need a prerequisite that the degree of signal sparsity *S* (the number of nonzero elements in **a**) is smaller than the number of measurements, *M*: $M\ge {\mathscr{O}}(S\,\log (N/S))$.

Conventional compressive sensing concerns the sparsity of the target **a**. In our study, the reflection matrix ${\boldsymbol{R}}\in {{\Bbb{C}}}^{N\times N}$ is sparsely sampled by the sensing matrix ${\boldsymbol{S}}\in {{\Bbb{C}}}^{N\times M}$: ***R***_S_ = ***RS***. Thus, the degree of sparsity of ***R*** is of concern. Essentially, our model ${\boldsymbol{R}}={{\boldsymbol{P}}}_{{\rm{o}}}{{\boldsymbol{O}}}_{{\rm{I}}}{{\boldsymbol{P}}}_{{\rm{i}}}^{{\rm{T}}}$ allows us to treat ***R*** as a highly sparse matrix. Since ***O***_I_ is diagonal and ${{\boldsymbol{P}}}_{{\rm{o}}({\rm{i}})}$ is a Toeplitz matrix, ***R*** contains only 3*N* unknowns. Since the measured ***R***_S_ contains the number of elements *N* × *M*, it forms a system of *N* × *M* equations with 3*N* unknowns. Theoretically, there needs $M\ge {\mathscr{O}}(3\,\log (N/3))$ to accurately estimate the solutions. However, we used the unknown speckle illuminations, i.e. unknown sensing matrix ***S***, and thus converted the problem to a decomposition of time reversal matrix, ${\boldsymbol{W}}={{\boldsymbol{P}}}_{{\rm{o}}}{{\boldsymbol{O}}}_{{\rm{I}}}{{\boldsymbol{P}}}_{o}^{\dagger }+{{\boldsymbol{W}}}_{{\rm{ms}}}+{{\boldsymbol{W}}}_{{\rm{\sigma }}}$, where ***W***_ms_ is due to multiple scattering noise, and ***W***_*σ*_ is the additional sparsity-induced noise. Since ***W***_*σ*_ scales with $1/\sqrt{M}$, the reduction of sampling *M* gives rise to the increase of noise. This makes it necessary to set *M* larger than the estimated value to properly decompose ***W***.

To identify the aberration and reconstruct an aberration-corrected image, the basis of ***W*** is changed to the spatial-frequency domain (**k**-space) by taking the Fourier transform for both the column and row bases. The CTR matrix in **k**-space is expressed as

5 $$\tilde{{\boldsymbol{W}}}={\tilde{{\boldsymbol{P}}}}_{{\rm{o}}}{\tilde{{\boldsymbol{O}}}}_{{\rm{I}}}{\tilde{{\boldsymbol{P}}}}_{o}^{\ast }+{\tilde{{\boldsymbol{W}}}}_{{\rm{N}}}+{\tilde{{\boldsymbol{W}}}}_{\sigma }$$

where a tilde (~) above the variable denotes the Fourier transformation of the variable into spatial-frequency domain. The matrix ${\tilde{{\boldsymbol{P}}}}_{{\rm{o}}}[{{\bf{k}}}_{{\rm{o}}}^{{\prime} };{{\bf{k}}}_{{\rm{o}}}]$ represents the planewave based transmission matrix between the object and output planes. When the output PSF is space-invariant, ${\tilde{{\boldsymbol{P}}}}_{{\rm{o}}}$ becomes a diagonal matrix whose elements are given by its complex pupil function ${\tilde{P}}_{{\rm{o}}}({{\bf{k}}}_{{\rm{o}}})={e}^{i{\phi }_{{\rm{o}}}({{\bf{k}}}_{{\rm{o}}})}$, where ${\phi }_{{\rm{o}}}({{\bf{k}}}_{{\rm{o}}})$ is the output pupil phase map. We consider here a phase-only pupil function that has amplitude of unity. The matrix ${\tilde{{\boldsymbol{O}}}}_{{\rm{I}}}$ is the target spectrum matrix in which each column consists of a shifted special-frequency spectrum of the target: ${\tilde{{\boldsymbol{O}}}}_{{\rm{I}}}[{{\bf{k}}}_{{\rm{o}}}^{{\prime} };{{\bf{k}}}_{{\rm{o}}}]={\tilde{O}}_{{\rm{I}}}[{{\bf{k}}}_{{\rm{o}}}^{{\prime} }-{{\bf{k}}}_{{\rm{o}}}]$.

The CTR-CLASS algorithm^10^, acting on the CTR matrix $\tilde{{\boldsymbol{W}}}$, identifies an aberration correction matrix ${\tilde{{\boldsymbol{P}}}}_{{\rm{c}}}$ that maximizes the total intensity of the object image ${\tilde{O}}_{\rm{I}}^{\rm{(c)}}({\bf{k}})$ reconstructed from a corrected CTR matrix ${\tilde{{\boldsymbol{W}}}}_{{\rm{c}}}={\tilde{{\boldsymbol{P}}}}_{{\rm{c}}}^{\ast }\tilde{{\boldsymbol{W}}}{\tilde{{\boldsymbol{P}}}}_{{\rm{c}}}$. Here, ${\tilde{O}}_{\rm{I}}^{\rm{(c)}}({\bf{k}})$ is reconstructed by the sum of ${\tilde{{\boldsymbol{W}}}}_{{\rm{c}}}$ along diagonals: ${\tilde{O}}^{\rm{(c)}}_{{\rm{I}}}({\bf{k}})={\sum }_{{{\bf{k}}}_{{\rm{o}}}}{\tilde{{\boldsymbol{W}}}}_{\rm{c}}[{{\bf{k}}}_{{\rm{o}}}+{\bf{k}};{{\bf{k}}}_{{\rm{o}}}]$. The algorithm iteratively finds the solution ${\tilde{P}}_{{\rm{c}}}({{\bf{k}}}_{{\rm{o}}})$ in the following way. At the *n*^th^ iteration, the *n*^th^ correction pupil function ${\tilde{P}}_{{\rm{c}}}^{(n)}({\bf{k}})={e}^{i{\phi }_{{\rm{c}}}^{(n)}({\bf{k}})}$, target spectrum ${\tilde{O}}_{\rm{I}}^{(n)}(\Delta {\bf{k}})$, and time-reversal matrix ${\tilde{{\boldsymbol{W}}}}^{(n)}$ are calculated as

6 $${\phi}_{\rm{c}}^{(n)}({{\bf{k}}}_{\rm{o}})={\mathrm{arg}}\left\{\sum _{\Delta {\bf{k}}\ne 0}{\tilde{W}}^{(n-1)}[{{\bf{k}}}_{{\rm{o}}}+\Delta {\bf{k}};{{\bf{k}}}_{{\rm{o}}}]\cdot {\tilde{O}}_{\rm{I}}^{(n-1)^\ast }(\Delta {\bf{k}})\right\}$$

7 $${\tilde{{\boldsymbol{W}}}}^{(n)}[{{\bf{k}}}_{{\rm{o}}}^{{\prime} };{{\bf{k}}}_{{\rm{o}}}]={\tilde{P}}_{{\rm{c}}}^{(n)}({{\bf{k}}}_{{\rm{o}}}^{{\prime} }){\tilde{{\boldsymbol{W}}}}^{(n-1)}[{{\bf{k}}}_{{\rm{o}}}^{{\prime} };{{\bf{k}}}_{{\rm{o}}}]{\tilde{P}}_{{\rm{c}}}^{(n)^\ast }({{\bf{k}}}_{{\rm{o}}})$$

8 $${\tilde{O}}_{I}^{(n)}(\Delta {\bf{k}})=\sum _{{{\bf{k}}}_{{\rm{o}}}}{\tilde{{\boldsymbol{W}}}}^{(n)}[{{\bf{k}}}_{{\rm{o}}}+\Delta {\bf{k}};{{\bf{k}}}_{{\rm{o}}}]$$

where $\Delta {\bf{k}}={{\bf{k}}}_{{\rm{o}}}^{{\prime} }-{{\bf{k}}}_{{\rm{o}}}$. The *n*^th^ correction phase angles ${\phi }_{{\rm{c}}}^{(n)}({{\bf{k}}}_{{\rm{o}}})$ is found by taking inner products of the angular spectrum images ${\tilde{W}}^{(n-1)}[{{\bf{k}}}_{{\rm{o}}}+\Delta {\bf{k}};{{\bf{k}}}_{{\rm{o}}}]$ and the corrected image ${\tilde{O}}_{\rm{I}}^{(n-1)}(\Delta {\bf{k}})$. Note that the target spectrum ${\tilde{O}}_{\rm{I}}^{(n)}(\Delta {\bf{k}})$ is reconstructed by synthesizing all the spatial frequency spectra covered by the input and output angles ($|{\bf{k}}/(2\pi )|$, $|{{\bf{k}}}_{{\rm{o}}}^{{\prime} }/(2{\rm{\pi }})|\le 2NA/\lambda$), resulting in a special-frequency band of $|\Delta {\bf{k}}/(2\pi )|\le 2{\rm{NA}}/\lambda$. The iteration starts with the initial conditions of ${\tilde{P}}_{{\rm{c}}}^{(0)}({{\bf{k}}}_{{\rm{o}}})=1$ and ${\tilde{{\boldsymbol{W}}}}^{(0)}=\tilde{{\boldsymbol{W}}}$ and continues until the root-mean-square (RMS) of the phase, ${{\rm{\sigma }}}_{\phi }^{2}={\langle {|{\phi }_{{\rm{c}}}^{(n)}({{\bf{k}}}_{{\rm{o}}})|}^{2}\rangle }_{{{\bf{k}}}_{{\rm{o}}}}$ becomes less than a predefined value. The final output correction phase function is found by accumulating all the preceding correction phases, ${\phi }_{{\rm{c}}}({{\bf{k}}}_{{\rm{o}}})={\sum }_{n=1}^{{n}_{{\mathrm{max }}}}{\phi }_{{\rm{c}}}^{(n)}({{\bf{k}}}_{{\rm{o}}})$.

### Experimental setup of CTR-CLASS microscopy

**Fig. 2:**
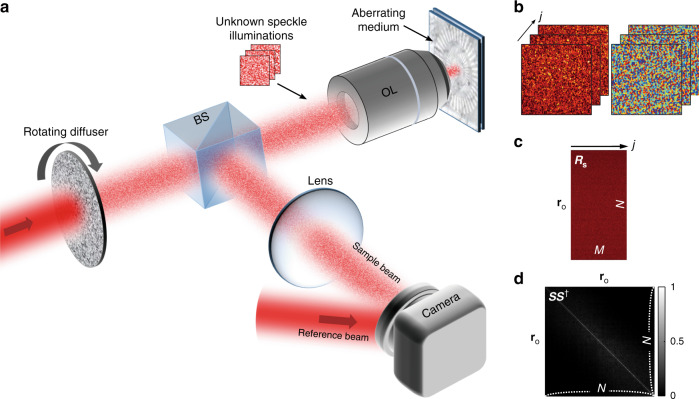
Experimental setup for recording a compressed time-reversal matrix. **a** Schematic of the experimental setup. BS: beamsplitter, OL: objective lens. A Ti: Sapphire pulsed laser is used as a low-coherence light source. Random speckle patterns produced by a rotating diffuser illuminate a sample. The off-axis holograms between the sample wave and a plane reference wave are recorded by a camera. Optics for reference beam path is omitted for brevity. **b** Representative E-field amplitude (left) and phase (right) images of the sample wave. The size of each image is 40 × 40 µm^2^, composed of *N* = 7744 orthogonal modes. **c** Sparsely-sampled timed-gated reflection matrix ***R***_S_ reconstructed by a set of *M* = 700 E-field images. **d** The matrix ***SS***^†^ obtained from an experimentally measured ***S*** by placing a mirror at the sample plane

The schematic of the experimental setup is shown in Fig. 2a for recording a CTR matrix ***W***. The basic configuration is a low-coherence wide-field interferometric microscope, but a sample is illuminated by random speckle fields while the reference wave is a planar wave. A custom-built wavelength-tunable Ti:Sapphire pulsed laser (center wavelength of 800–900 nm, bandwidth of 30 nm) was used as a low-coherence light source. An optical diffuser mounted on a motorized rotation stage was inserted at a conjugate image plane in the illumination path to produce uncorrelated random speckle fields for the sample wave. Backscattered sample waves from the target were captured by an objective lens (Nikon, ×60, NA 1.0) and delivered to a high-speed CMOS camera (Photron, FASTCAM mini UX100) placed at a conjugate image plane. A reference plane wave was introduced at the camera to generate the off-axis low-coherence interferogram, from which we obtained the time-gated E-field of the backscattered sample wave (see Supplementary Information Note 1 for the detailed setup). Figure 2b shows three representative E-field amplitude and phase images of the sample under different speckled illuminations. To obtain a complete *N*-by-*N* reflection matrix for a FOV having *N* orthogonal modes, *N* E-field images must be acquired for an orthogonal set of *N* illumination fields, where each image has a total of *N* orthogonal pixels. However, we recorded only *M* (<*N*) E-field images using random speckle illuminations to reduce the acquisition time and reconstruct a sparsely sampled *N*-by-*M* reflection matrix ***R***_S_ as shown in Fig. 2c.

For efficient sparse sampling of the reflection matrix, it is important to minimize the correlation between the speckle fields as much as possible. For a given camera exposure time and frame rate, the angular velocity of the rotating diffuser was carefully selected to minimize the spatial correlation between two consecutive speckle patterns (Supplementary Information Note 2). To justify the validity of ***SS***^†^ **≈** ***I***, we experimentally measured the **S** matrix by placing a mirror at the sample plane. Figure 2d shows ***SS***^†^ matrix obtained using *M* = 700 speckled illuminations for a FOV of 40 × 40 µm^2^ with a diffraction-limited resolution of 450 nm, resulting in the total number of orthogonal modes, *N* = 88 × 88 = 7744. The **S*S***^†^ was nearly diagonal matrix with the ratio between off-diagonal and diagonal elements of ≲0.1.

### Proof-of-concept experiment

To demonstrate the high-throughput data acquisition and aberration correction capabilities of the CTR-CLASS microscopy, we imaged a homemade Siemens star target covered by a 600-µm-thick plastic layer introducing strong optical aberrations. The laser operated at a center wavelength of 900 nm, and had a coherence length of ~12 µm. For a FOV of 40 × 40 µm^2^ (*N* = 88 × 88 pixels), *M* speckled E-field images of the target were imaged by the high-speed camera operating at a frame rate of 12,500 Hz with an exposure time of 20 µs.

**Fig. 3:**
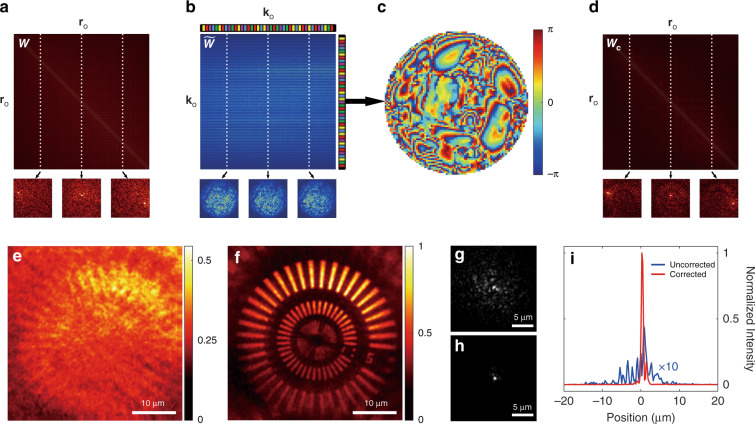
Wavefront aberration correction using time-reversal matrix. **a** Fully-sampled time-reversal matrix ${\boldsymbol{W}}={{\boldsymbol{R}}}_{\rm{S}}{{\boldsymbol{R}}}_{\rm{S}}^{\dagger }$ (*CR* = 1) for a Siemens star resolution target underneath a highly aberrating medium. **b** Time-reversal matrix $\tilde{{\boldsymbol{W}}}$ in k-space, obtained by the Fourier transform of ***W***. **c** Wavefront aberration map *ϕ*_o_(**k**_**o**_***k***) at the output pupil plane, identified by the CLASS algorithm. Color bar indicates the phase in radians. **d** Aberration-corrected time-reversal matrix ***W***_*c*_. The subset images in (**a**, **b**, and **d**) are 2D E-field images corresponding to columns marked by dashed lines. **e**, **f** Uncorrected and corrected images of the Siemens star reconstructed from $\tilde{{\boldsymbol{W}}}$ and ${\tilde{{\boldsymbol{W}}}}_{{\rm{c}}}$, respectively. Images are normalized by the maximum intensity in the corrected image. **g**, **h** Uncorrected and corrected intensity-PSFs, respectively. **i** Line profiles of the uncorrected and corrected intensity-PSFs in (**g**, **h**)

We define the compression ratio that indicates the degree of sparse sampling as *CR* = *M*/*N*. Figure 3a shows the CTR matrix ***W*** constructed by speckle patterns for *CR* = 1. In the absence of aberration (***P***_o_ = ***I***), the matrix ${\boldsymbol{W}}\approx {{\boldsymbol{P}}}_{{\rm{o}}}{{\boldsymbol{O}}}_{{\rm{I}}}{{\boldsymbol{P}}}_{o}^{\dagger }$ is almost diagonal because it is reduced to ***O***_I_. In the presence of aberration and scattering, the signal in the diagonal spreads out to the off-diagonal elements. The matrix $\tilde{{\boldsymbol{W}}}$ in spatial-frequency domain and the identified correction phase ${\phi }_{{\rm{c}}}({{\bf{k}}}_{{\rm{o}}})$ by the CTR-CLASS algorithm are shown in Fig. 3b, c, respectively. The corrected CTR matrix ${{\boldsymbol{W}}}_{{\rm{c}}}={{\boldsymbol{P}}}_{{\rm{c}}}^{\dagger }{\boldsymbol{W}}{{\boldsymbol{P}}}_{{\rm{c}}}$ is shown in Fig. 3d. The intensity images before and after the aberration correction were reconstructed from $\tilde{{\boldsymbol{W}}}$ and ${\tilde{{\boldsymbol{W}}}}_{{\rm{c}}}$, respectively. The uncorrected image shown in Fig. 3e is blurry and hardly recognizable, while object structures are sharply resolved in the corrected image in Fig. 3f. Both the imaging resolution and the signal-to-background ratio (SBR) were significantly improved compared to those in the uncorrected image. We quantified the degree of aberration correction by measuring the normalized intensity profiles of PSFs before and after the aberration correction (Fig. 3g–i). The Strehl ratio, defined by the peak intensity of the PSF, is a measure of AO performance. The Strehl ratio *α*_o_ before aberration correction is given by ${\alpha }_{{\rm{o}}}={|{P}_{{\rm{o}}}(0)|}^{2}$. Likewise, the Strehl ratio *α*_c_ after correction is given by ${\alpha }_{{\rm{c}}}={|{P}_{{\rm{res}}}(0)|}^{2}$, where *P*_res_(**r**) is the residual PSF after the correction (${{\boldsymbol{P}}}_{{\rm{res}}}={{\boldsymbol{P}}}_{{\rm{c}}}^{\dagger }{{\boldsymbol{P}}}_{{\rm{o}}}$). The enhancement in the Strehl ratio, (*α*_c_/*α*_o_) measured from the line profiles of the PSFs was at least 20. The measured full-width at half-maximum (FWHM) of the aberration-corrected PSF was about 450 nm, which is the diffraction-limit spatial resolution of the system.

### Analysis of image SNR

**Fig. 4:**
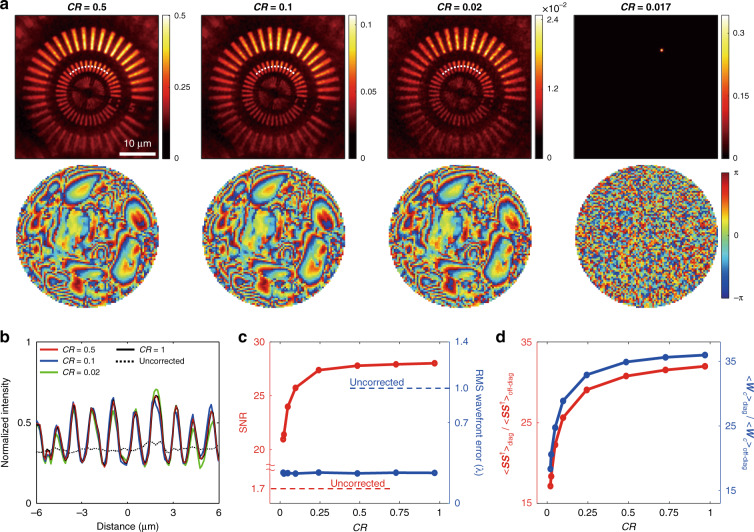
Image reconstruction fidelity depending on the compression ratio (*CR*). **a** Aberration-corrected images (upper row) and corresponding aberration maps (lower rows) for various *CR* values. **b** Line profiles along the white dashed lines in (**a**). **c** SNR (red dots) and residual RMS wavefront error (blue dots) depending on *CR*. **d** Ratio between the averaged diagonal elements and off-diagonal elements of ***SS***^†^ (red dots) and ***W***_c_ (blue dots) depending on *CR*. The solid curves in (**c**, **d**) are linear interpolations of the data

We evaluated the performance of image recovery depending on *CR* to determine the minimum achievable *CR*. Reconstructed images and aberration phase maps for various *CR* are shown in Fig. 4a. The first row shows the reconstructed intensity images normalized by *M* for *CR* = 0.5, 0.1, 0.02, and 0.017, and the second row shows the corresponding aberration phase maps. The identified aberration maps were almost identical all the way into the high spatial frequency range, although *CR* was significantly reduced. Diffraction-limited high-resolution images could be successfully restored for *CR* ≥ 0.02 (*M* ≥ 155). Considering the camera frame rate of 12,500 Hz, it took only 12.4 ms to record the CTR matrix ***W*** for *CR* = 0.02, setting the highest achievable aberration-correction image frame rate to 80 Hz for a FOV of 40 × 40 µm^2^ (88 × 88 pixels). If the *CR* was further reduced to less than 0.02, the image reconstruction failed to find the correct aberration map and object image. The line profiles along the white dotted lines on the reconstructed images in Fig. 4a are compared in Fig. 4b to quantify the image quality. Interestingly, neither the image contrast nor the spatial resolution of the reconstructed image was diminished by the reduction of *CR*, so far as the image reconstruction was successful. This means that even the severe decrease in *CR* does not hamper the performance of aberration correction. We compared the residual root-mean-square (RMS) wavefront errors of the identified wavefront maps relative to the aberration map obtained from the full reflection matrix (blue dots in Fig. 4c). The residual RMS wavefront errors remained nearly constant regardless of *CR*. This indicates that aberrations were properly corrected even with the small *M*. The pupil phase retardation ${\phi }_{{\rm{c}}}^{(n)}({{\bf{k}}}_{{\rm{o}}})$ is estimated from the inner product of the angular spectrum image for the illumination angle of **k**_o_, $\tilde{W}[{{\bf{k}}}_{{\rm{o}}}+\Delta {\bf{k}};{{\bf{k}}}_{{\rm{o}}}]$ and the angular synthesized image ${\tilde{O}}_{{\rm{I}}}(\Delta {\bf{k}})$ in Eq. (6), where the summation is taken over total *N* spectral frequencies, ∆**k**. Thus, the standard error of the estimated pupil phase scales with $1/\sqrt{N}$. Essentially, the performance of aberration correction is determined by *N*, the number of sampled spectral frequencies or equivalently the image size. Therefore, as long as *N* is sufficiently large, the pupil phase retardation can be identified with high fidelity even for a small number of measurements, *M*.

An important figure of merit in imaging is the signal-to-noise ratio (SNR), which is defined by the ratio of the mean intensity of the target image and the standard deviation of random background noise. To estimate the image SNR, we consider the aberration-corrected CTR matrix ${{\boldsymbol{W}}}_{{\rm{c}}}={{\boldsymbol{P}}}_{{\rm{c}}}^{\dagger }{\boldsymbol{W}}{{\boldsymbol{P}}}_{{\rm{c}}}={{\boldsymbol{P}}}_{{\rm{c}}}^{\dagger }{{\boldsymbol{P}}}_{{\rm{o}}}{{\boldsymbol{O}}}_{{\rm{I}}}{{\boldsymbol{P}}}_{o}^{\dagger }{{\boldsymbol{P}}}_{{\rm{c}}}+{{\boldsymbol{P}}}_{{\rm{c}}}^{\dagger }{{\boldsymbol{W}}}_{{\rm{ms}}}{{\boldsymbol{P}}}_{{\rm{c}}}+{{\boldsymbol{P}}}_{{\rm{c}}}^{\dagger }{{\boldsymbol{W}}}_{{\rm{\sigma }}}{{\boldsymbol{P}}}_{{\rm{c}}}$. Note that the incident speckle field is normalized as ${\langle {\sum }_{j=1}^{M}{|S({{\bf{r}}}_{{\rm{i}}};j)|}^{2}\rangle }_{{{\bf{r}}}_{{\rm{i}}}}=1$, which means the average intensity of speckle illumination fields at each pixel **r**_i_ is 1/*M*. For intuitive understanding, let us consider sending speckle illumination fields whose average intensity per pixel is 1. We define the signal *s*_c_ as the intensity of the target reconstructed after the correction, which corresponds to the main diagonal of the first term ${{\boldsymbol{P}}}_{{\rm{c}}}^{\dagger }{{\boldsymbol{P}}}_{{\rm{o}}}{{\boldsymbol{O}}}_{{\rm{I}}}{{\boldsymbol{P}}}_{o}^{\dagger }{{\boldsymbol{P}}}_{{\rm{c}}}$. Therefore, *s*_c_ is given by ${s}_{{\rm{c}}}=M{\alpha }_{{\rm{c}}}{O}_{{\rm{I}}}({\bf{r}})$. There are mainly two kinds of noise sources: (1) measurement noise ***W***_ms_ and (2) sparse sampling-induced noise ***W***_σ_. First, the measurement noise includes multiple scattering noise, photon shot noise, and dark count noise of a camera sensor. In the case of imaging through a scattering medium, the measurement noise is dominated by multiple scattering noise, which is much larger than photon shot noise and dark count noise. Due to the random nature of noise, the standard deviation of the measurement noise, *σ*_ms_ remains almost unchanged after aberration correction and is proportional to the square root of *M*: ${\sigma }_{{\rm{ms}}}=\sqrt{M}m$. Here *m* denotes the measurement noise in a single wide-field image, which is mainly given by the average intensity of multiple scattering noise. Second, as we discussed earlier, the sparse sampling-induced noise is inherently caused by the overlaps among speckle illumination patterns and is given by ${{\boldsymbol{W}}}_{\sigma }={{\boldsymbol{P}}}_{{\rm{o}}}{\boldsymbol{O}}{\boldsymbol{\sigma }}{{\boldsymbol{O}}}^{\dagger }{{\boldsymbol{P}}}_{o}^{\dagger }$. The standard deviation of the sparse sampling-induced noise, σ_s_ also scales proportionally with the square root of *M*: ${\sigma }_{{\rm{s}}}=\sqrt{M}{\langle {O}_{{\rm{I}}}({\bf{r}})\rangle }_{{\bf{r}}}$, where ${\langle {O}_{{\rm{I}}}({\bf{r}})\rangle }_{{\bf{r}}}$ is the average reflectance of the target. In fact, the *σ*_s_ is analogous to the measurement noise, except that it is proportional to the target’s average reflectance. Then, the SNR_c_ can be estimated to be ${{\rm{SNR}}}_{{\rm{c}}}={s}_{{\rm{c}}}/\sqrt{{\sigma }_{{\rm{ms}}}^{2}+{\sigma }_{{\rm{s}}}^{2}}$.

In the strong multiple scattering regime (${O}_{{\rm{I}}}({\bf{r}})\ll m$), the SNR_c_ of the reconstructed image from the CTR matrix is approximated as ${{\rm{SNR}}}_{{\rm{c}}}\approx {s}_{{\rm{c}}}/{\sigma }_{{\rm{ms}}}=\sqrt{M}({\alpha }_{{\rm{c}}}/{\alpha }_{{\rm{o}}}){{\rm{SNR}}}_{1}$, where ${{\rm{SNR}}}_{1}={\alpha }_{{\rm{o}}}{O}_{{\rm{I}}}({\bf{r}})/m$ is the SNR of a single wide-field image obtained without correction. In the weak scattering regime of SNR_1_ ≫ 1, the SNR_c_ is mainly determined by the sparse sampling-induced noise and given by ${{\rm{SNR}}}_{{\rm{c}}}\approx {s}_{{\rm{c}}}/{\sigma }_{{\rm{s}}}=\sqrt{M}{\alpha }_{{\rm{c}}}$(${O}_{{\rm{I}}}({\bf{r}})/{\langle {O}_{{\rm{I}}}({\bf{r}})\rangle }_{{\bf{r}}}$). In this proof-of-concept experiment, the plastic aberrating layer caused strong aberrations, but not much the multiple scattering noise. The Siemens star target used in this demonstration consists of binary patterns with nearly 100% reflectivity (${O}_{{\rm{I}}}({\bf{r}})=1$) and a fill factor value of ~50% (${\langle {O}_{{\rm{I}}}({\bf{r}})\rangle }_{{\bf{r}}}=1/2$), giving the ratio ${O}_{{\rm{I}}}({\bf{r}})/{\langle {O}_{{\rm{I}}}({\bf{r}})\rangle }_{{\bf{r}}}\approx 2$. In this specific case of a binary target with 50% fill factor, the SNR_c_ is expressed as ${\rm{SNR}}=2\sqrt{M}{\alpha }_{{\rm{c}}}$. In terms of compression ratio, it is expressed as ${\rm{SNR}}=2\sqrt{N}{\alpha }_{{\rm{c}}}\sqrt{CR}$. We plotted the SNR as a function of *CR* in Fig. 4c (red dots) and observed that the SNR fits well with $\sqrt{CR}$. The ratio between the diagonal and off-diagonal elements of ***W***_c_ is the signal to background noise ratio, which in turn is equal to the SNR of the reconstructed image. We found that ratios between the diagonal to off-diagonal elements of both ***SS***^†^ and ***W***_c_ also fit well with $\sqrt{M}$ as shown in Fig. 4d, confirming the validity of our analysis. We found that this ratio fits well with $\sqrt{M}$, confirming the validity of our SNR analysis.

Image reconstruction was successful up to a *CR* value of 0.02 (*M* ≥ 155), but it was shown to fail with a further decrease of *CR*. As explained above, the reduction in *CR* introduces random noise. Below a certain threshold *CR* value, the noise level becomes too high for the algorithm to retrieve the pupil phase map. It is difficult to generalize the minimum achievable *CR* value because it depends on various factors such as the SNR_1_ of the uncorrected image, the distribution of the target spectrum, and the complexity of the aberration. Therefore, appropriate choice of *CR* value is necessary depending on the sample for an optimal image acquisition speed and quality.

### Volumetric aberration-correction imaging of a mouse brain

**Fig. 5:**
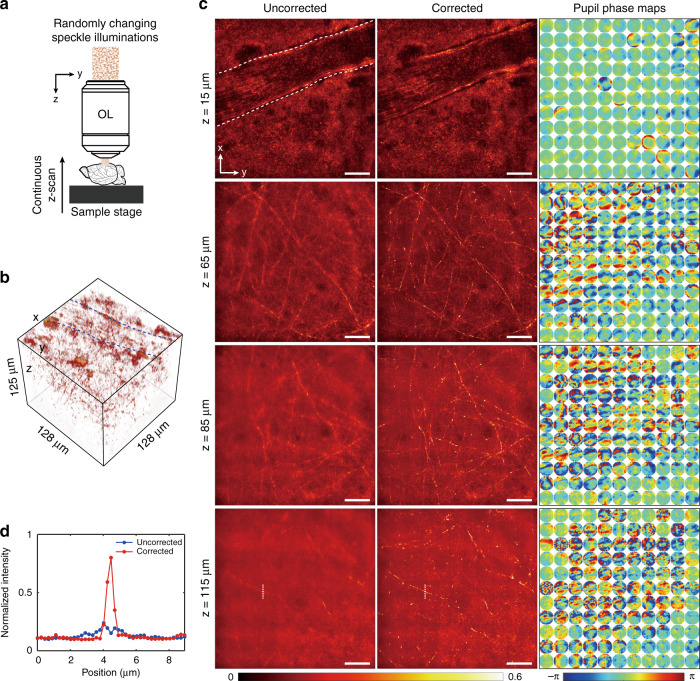
Aberration-free volumetric high-speed imaging of a mouse brain. **a** Imaging configuration. The mouse brain tissue was continuously scanned along the *z*-axis at a constant speed while dynamically varying speckle patterns illuminated the specimen. **b** Reconstructed 3D volumetric image of brain tissue with transverse field of view of 128 × 128 µm and depth range of 125 µm, composed of 568 × 568 × 125 voxels. The two white dashed curves indicate wall boundaries of a blood vessel with a diameter of ~30 µm, located close to the brain surface. **c** Left-hand and middle columns show MIP images with and without aberration correction at various depths, respectively. MIP range: 6 µm for *z* = 15, 65, and 85 µm, and 20 µm for *z* = 115 um. The right-hand column shows reconstructed output pupil phase maps for 11 × 11 subregions. The white dashed curves in the uncorrected image at *z* = 15 µm indicate wall boundaries of a blood vessel near the brain surface. The radius of each circle corresponds to a numerical aperture of NA = 0.94. The images at each depth were normalized by the maximum intensity in the corrected image. Scale bar: 20 µm. **d** Line profiles along the vertical white dotted lines in the images at *z* = 115 µm in (**b**)

With the CTR-CLASS microscope, we demonstrated the high-throughput aberration-corrected volumetric imaging of myelinated axons in an ex vivo mouse brain. In this demonstration, the pulsed laser operated at a center wavelength of 848 nm with a coherence length of 8 µm. Typically, a set of E-field images constituting a reflection matrix are recorded for each fixed depth to maintain the input and output planes throughout the measurements. This depth-wise matrix recording slows down the volumetric imaging. Here, we employed continuous depth scanning to speed up the volume coverage. Since the output planes are continuously varying, we added numerical propagation steps to synchronize the output planes prior to the application of CTR-CLASS algorithm. The synchronization of the input planes is not necessary as there is no need to know the used input channels. To experimentally implement this concept, a whole mouse brain was mounted on a motorized stage and continuously scanned along the *z*-axis at a constant speed of 35 µm/s while dynamically varying speckle patterns illuminated the specimen (Fig. 5a). The E-field images of the backscattered waves were recorded by the high-speed camera at a frame rate of 5000 Hz. Therefore, there was 7 nm depth difference between the neighboring frames. The camera exposure time (50 µs) and the angular velocity of the rotating diffuser (210 degree/s) were carefully selected to ensure that the spatial correlation of the speckle patterns between the consecutive frames was less than 0.1 (See Supplementary Information Note 2). For the total image acquisition time of 3.58 s, we obtained a series of 17,891 E-field images with a frame size of 128 × 128 µm^2^ (300 × 300 pixels), spanning a depth range of 125 µm from the surface of the brain. The angular spectra of the obtained E-field images are filtered by applying a binary pupil mask with NA = 0.94. Then, we finally obtained E-field images with a size of 128 × 128 µm^2^ (284 × 284 pixels) and diffraction-limited resolution of 0.45 nm.

To reconstruct a 3D volume image, we first prepared depth-corrected E-field images at 125 depths spaced 1 µm apart. To retrieve an aberration-corrected image at each given depth *z*, E-field images taken within a range of *z* ± 4 µm numbering 1147 were numerically propagated to the depth z by adding appropriate quadratic spectral phase factors in their angular spectra. To deal with position-dependent aberrations, the depth-corrected E-field images were divided into 11 × 11 subregions, and the CTR matrix for each subregion was separately constructed using these images. Finally, we retrieved aberration-corrected 2D images by applying the CLASS algorithm to the CTR matrices at individual subregions in all depth *z*. The reconstructed 3D volume image over 128 × 128 × 125 µm^3^ (568 × 568 × 125 voxels) is shown in Fig. 5b (See Supplementary Video 1). Note that the depth-dependent defocus due to refractive index mismatch between immersion water and the tissue causes the separation of the objective focus and coherence volume resulting in blurred images^28^. We could find and compensate the depth-dependent defocus by numerically propagating the E-field images such that the total intensity of reconstructed images without aberration correction was maximized. The depth-dependent defocus was about 3 µm at *z* = 100 µm, which was less than the coherence length (8 µm) of light source. Representative section images at various depths are shown in Fig. 5c. The left-hand column shows maximum intensity projection (MIP) of the reconstructed images without aberration correction, whereas the middle column shows corresponding MIP images after aberration correction. The right-hand column shows identified output pupil phase maps for 11 × 11 subregions. There were no significant aberrations at *z* = 15 µm except for spherical aberration. Up to a depth of 50 µm, the aberration-corrected images were almost identical to those without correction. As the imaging depth was increased, the aberration maps became more complex, and myelinated fibers in the uncorrected images became blurred due to the inhomogeneity within the tissue. Specifically, there was a blood vessel with a diameter of ~30 µm located close to the surface of the brain. The white dashed curves in the uncorrected image at *z* = 15 µm in Fig. 5c indicate wall boundaries of the blood vessel. We observed that the blood vessel induced pronounced aberrations such that the aberration maps in areas under the vessel were more complex than those in other areas. In addition, correlation between aberration maps of neighboring subregions decreased rapidly, suggesting that the isoplanatic patch size was reduced with depth. At the depth of 115 µm, myelinated axons were almost invisible without aberration correction. Intensity line profiles along the white dotted lines in the images at *z* = 115 µm are shown in Fig. 5d. Comparing the line profiles, we observed that the CTR-CLASS can recover a nearly diffraction-limit resolution of ~0.45 µm (the minimum thickness of myelinated fiber in FWHM) and high-contrast images of myelinated fibers (up to ~7-fold increase in signal-to-background ratio). The axial resolution measured from cross-sections of myelin fibers along *z*-axis was ~2 µm.

## Discussion

The reflection matrix containing full optical input–output response of a scattering medium has offered robust image reconstruction in comparison with conventional adaptive optics approaches relying on partial information. In particular, it enables the correction of extremely complex aberrations in stringent conditions where there are strong multiple scattering noise and no guide stars available. As a trade-off, the matrix recording is too time-consuming to perform real-time imaging. Throughout our study, we demonstrated that the use of a time-reversal matrix, instead of the reflection matrix, can be a solution for the high-throughput volumetric imaging equipped with all the benefits of the reflection matrix approaches. We proved that the time-reversal matrix approach can maintain the fidelity of aberration correction and image reconstruction using as small as 2% of the full basis sampling. Due to nearly 100-fold reduction of the matrix recording time, we could achieve aberration-correction imaging for a 2D FOV of 40 × 40 µm^2^ at a frame rate of 80 Hz. Furthermore, we realized the volumetric imaging of a mouse brain over a volume of 128 × 128 × 125 µm^3^ in 3.58 s with a lateral resolution of 0.45 µm and an axial resolution of 2 µm throughout all the voxels including the areas underneath a blood vessel.

The proposed method presents a noteworthy conceptual advance. It is a new discovery that the time-reversal matrix can be highly compressed in terms of illumination channel coverage. We found that it is not even necessary to know what the illumination channels were. These conceptual findings naturally led to the advances in practicality. In addition to the reduction of illumination channel coverage, there is no need to perform time-consuming pre-calibration to gain prior knowledge on illumination field. It is no longer necessary to concern the phase stability among the E-field images. This enabled us to use dynamically varying random speckle patterns for illumination, instead of laser beam scanning by carefully aligned scanning mirrors, which greatly simplifies the experimental setup. We also presented novel volumetric image processing algorithm that replaces previous depth-wise angular scanning with continuous depth scanning in conjunction with dynamic speckle illuminations. We introduced the depth-correction step where all E-field images taken at different depths within the coherence length of the light source were numerically propagated to the target depth. This increases the number of images to be used for constructing a time-reversal matrix at each target depth, which effectively increases the volumetric imaging speed.

All these benefits of using the compressed time-reversal matrix come with a price to pay. A finite overlap between random illumination channels introduces additive noise in addition to multiple scattering noise. Therefore, achievable imaging depth is reduced relative to the full sampling by the amount of sparse sampling-induced noise. Using orthogonal illumination channels such as the Hadamard patterns instead of unknown speckles can minimize the sparse sampling-induced noise at the expense of hardware simplicity. In case when a priori knowledge of the scene is known, the number of required measurements could be drastically reduced by introducing a learned sensing approach^29,30^ using optimized illumination channels. Another drawback is that the achievable imaging resolution with the CTR-CLASS algorithm is diffraction limited. This is because, without knowledge of the illumination channels, the spatial cut-off frequency is solely determined by that of detection channels. The above shortcoming can be overcome by introducing a new image reconstruction algorithm combining the CTR-CLASS with methods that can reconstruct super-resolution images without prior knowledge of the illumination patterns, such as blind structured illumination microscopy^31^ and random illumination microscopy^32,33^. In this study, ballistic waves scattered once by an object are used for image reconstruction, and multiple-scattered waves inside a scattering medium are considered as background noise. However, multiple-scattered waves do also carry spatial information of the object. CTR-CLASS algorithm can potentially be extended to make the deterministic use of multiple-scattered waves in image reconstruction for further reducing measurement time or lowering the achievable spatial resolution well below the diffraction limit^34^.

High-throughput volumetric imaging equipped with aberration correction capability for every depth section allows detailed mapping of microstructures deep within tissues. This will lead to accurate quantification of structural and molecular information in various biological systems. Therefore, the presented method will find its use for a wide range of studies in life science and medicine including the myelin-associated physiology in neuroscience, retinal pathology in ophthalmology and endoscopic disease diagnosis in internal organs. Due to the high-speed measurement of tissue aberration, it can also serve as wavefront sensing AO to provide aberration information for the hardware aberration correction. This will help to improve the imaging depth of fluorescence and nonlinear imaging modalities such as multi-photon microscopy, super-resolution microscopy, and coherent Raman microscopy.

## Materials and methods

### Acquisition time for the CTR-matrix

As long as the laser power is enough, the major factor determining the acquisition of the CTR-matrix is the frame rate of the camera, *f*_cam_. In this study, a laser power of about 40 mW was illuminated on mouse brain sample for volumetric imaging, which was sufficient to obtain high SNR camera images with an exposure time of 50 µs. The acquisition time set by the frame rate of the camera is given by *T* = *M*/*f*_cam_. The size of the camera pixel corresponds to 0.128 µm in sample space. In the proof-of-concept experiment with a phantom aberrating layer, a series of camera images (1280 × 400 pixels) were acquired with a frame rate of 12,500 Hz and cropped to 312 × 312 pixels, which corresponds to a FOV of 40 × 40 µm^2^ in sample space. For volumetric imaging of the mouse brain, a series of camera images (1280 × 1000 pixels) were acquired with a frame rate of 5000 Hz, which corresponds to a FOV of 164 × 128 µm^2^ in sample space.

### Animal preparation

Adult (over 8 weeks) C57BL/6 mice were deeply anesthetized with an intraperitoneal injection of ketamine/xylazine (100/10 mg/kg) and decapitated. After the scalp and skull were removed, the brain was fixed with 4% paraformaldehyde at 4 °C overnight and washed with phosphate-buffered saline (PBS) three times. For imaging, the fixed brain was stuck to a plastic dish and immersed in PBS. All animal experiments were approved by the Korea University Institutional Animal Care & Use Committee (KUIACUC-2019-0024).

## Supplementary information

Supplementary information

Supplementary video 1

## Data Availability

All relevant data are available from the authors upon request.
