# Supplementary material for: High-throughput volumetric adaptive optical imaging using compressed time-reversal matrix
Source: Light Sci Appl. 2022 Jan 14;11:16. doi: 10.1038/s41377-021-00705-4 (PMC8758712; doi:10.1038/s41377-021-00705-4)
Supplement: Supplementary file 1 — Supplementary information [file 41377_2021_705_MOESM1_ESM.docx]

**Supplementary Information for High-throughput volumetric adaptive optical imaging using compressed time-reversal matrix**

Hojun Lee^1,2,+^, Seokchan Yoon^1,2,+^, Pascal Loohuis^3,4^, Jin Hee Hong^1^, Sungsam Kang^1,2^, and Wonshik Choi^1,2,*^

*^1^Center for Molecular Spectroscopy and Dynamics, Institute for Basic Science, Seoul 02841, Korea*

*^2^Department of Physics, Korea University, Seoul 02841, Korea*

*^3^**Department of Applied Mathematics, University of Twente, Drienerlolaan 5, 7522 NB Enschede, Netherlands*

*^4^Achmea Sz Holding BV,* *Handelsweg 2, 3707 NH Zeist, Netherlands*

^+^*These authors contributed equally to this work.*

*^*^wonshik@korea.ac.kr*

# Note I: Detailed experimental setup

Delay

stage

Ti:Sapphire

pulsed laser

HWP1

sample

0.8

PBS

HWP2

L1

L2

RD

L3

L4

L5

L6

Grating

POL

Camera

L7

L8

L9

BS

OL

**Fig. S1. Details of experimental setup.** HWP1-2: half wave plate, PBS: polarizing beam splitter, BS: beam splitter, POL: linear polarizer, OL: objective lens, RD: Rotating diffuser. L1-8: lens

We designed a reflection-type interferometric microscopy system to measure the electric field signals scattered from the target. A detailed schematic of the setup is shown in Fig. S1. A Ti:Sapphire pulsed laser (output power of 1.2 W, repetition rate of 80 MHz, central wavelength of 800 to 900 nm, pulse duration of < 100 fs) is used as a low-coherence light source to realize a temporal gating. The HWP1 and PBS1 are used to split the laser beam into the sample beam (red line) and reference beam (blue line). The HWP2 rotates the polarization of the reference beam so that the two beams can interfere at the camera. In the sample arm, a lens pair (L1 and L2) is installed to expand the diameter of the Gaussian-shaped laser beam to 4 mm in full width at half maximum (FWHM) at a rotating diffuser located at a conjugate image plane. The speckle field generated by the diffuser is then properly magnified by a lens pair (L3 and L4) to completely fill the objective rear aperture and imaged onto the sample plane by a tube lens (L5) and the objective lens (OL). The magnification factor from the diffuser plane to the sample plane is 1/32. The backscattered wave from the sample is picked up by BS and detected by a high-speed camera (10-μm pixel pitch, FASTCAM mini UX100, Photron) placed on the conjugate image plane. The sample plane is imaged on the camera using two pairs of lenses (OL-L5 and L4-L6) with a total magnification factor of 77. We used a planewave as a reference wave to measure the amplitude and phase of the sample speckle fields by means of standard off-axis digital holography. The first-order diffraction of the reference beam generated by a grating (Ronchi Ruling, 72 lp/mm, Edmund Optics) is sent to the camera to interfere with the sample field.

# Note II: Determination of diffuser rotational speed

**a**

**b**

Speckle number, *j*

1

4

8

12

16

1

0.5

0

Speckle number, *j*

Speckle number, *j*

*ω* = 60 deg/s

*ω* = 100 deg/s

*ω* = 200 deg/s

Cross correlations

0.65

0.37

0.1

*ω*

1

4

8

12

16

1

4

8

12

16

1

0.5

0

1

0.5

0

*r*

**Fig. S2. Speckle correlations vs. diffuser rotational speed.** **a** The geometry of the laser beam incident on the rotating diffuser. **b** Normalized cross correlations between the first and *j*^th^ speckle fields for various *ω*. The camera frame rate was 5,000 Hz, and the exposure time was 50 µs. The correlation between two consecutive speckle fields, indicated by the red arrows are about 0.65, 0.37, and 0.1 for *ω* = 60, 100, and 200 deg/s, respectively.

A diffuser made of a sheet of parafilm (thickness: 140 µm, diameter: 85 mm, Parafilm M, Bemis Company) was mounted on a motorized rotation stage (DDR25, Thorlabs). A laser beam of diameter 4 mm in FWHM passes through the diffuser at a position *r* ≈ 35 mm from the center of the diffuser (Fig. S2a). To generate dynamic speckle fields, the diffuser is continuously rotated with a constant rotational speed *ω* [deg/s]. To investigate the relationship between the rotational speed and the correlation between speckles, we obtained speckle E-field images $S\left( \mathbf{r};j \right)$ with a mirror at the sample plane. The normalized correlations between the first and *j*^th^ speckle fields, $C(j)=\frac{\left\langle S\left( \mathbf{r};j \right)S^{*}\left( \mathbf{r};1 \right) \right\rangle_{\mathbf{r}}}{\sqrt{\left\langle\left| S\left( \mathbf{r};j \right) \right|^{2} \right\rangle_{\mathbf{r}}\left\langle\left| S\left( \mathbf{r};1 \right) \right|^{2} \right\rangle_{\mathbf{r}}}}$ were calculated for various rotational speed. Figure S2b shows the $C(j)$ for a camera frame rate of 5,000 Hz and an exposure time of 50 µs. When *ω* ≥ 200 deg/s, the correlation between two consecutive speckle fields $C(2)$ falls below 0.1, ensuring the condition $\mathbf{S}\boldsymbol{S}^{\boldsymbol{\dagger}}\boldsymbol{\approx I}$. In the first experiment presented in Fig. 3 and 4, the rotational speed of *ω* = 540 deg/s was used for a camera frame rate of 12,500 Hz and an exposure time of 20 µs. In the second experiment presented in Fig. 5, the rotational speed of *ω* = 210 deg/s was used for a camera frame rate of 5,000 Hz and an exposure time of 50 µs.

# Note III: Supplementary video 1

The supplementary video 1 provided in the supplementary information shows the aberration-corrected 3D image of the mouse brain for a volume of 128×128×125 µm^3^ (568×568×125 voxels).
